# Denitrification in Agriculturally Impacted Streams: Seasonal Changes in Structure and Function of the Bacterial Community

**DOI:** 10.1371/journal.pone.0105149

**Published:** 2014-08-29

**Authors:** Erin Manis, Todd V. Royer, Laura T. Johnson, Laura G. Leff

**Affiliations:** 1 Department of Biological Sciences, Kent State University, Kent, Ohio, United States of America; 2 School of Public and Environmental Affairs, Indiana University, Bloomington, Indiana, United States of America; Missouri University of Science and Technology, United States of America

## Abstract

Denitrifiers remove fixed nitrogen from aquatic environments and hydrologic conditions are one potential driver of denitrification rate and denitrifier community composition. In this study, two agriculturally impacted streams in the Sugar Creek watershed in Indiana, USA with different hydrologic regimes were examined; one stream is seasonally ephemeral because of its source (tile drainage), whereas the other stream has permanent flow. Additionally, a simulated flooding experiment was performed on the riparian benches of the ephemeral stream during a dry period. Denitrification activity was assayed using the chloramphenicol amended acetylene block method and bacterial communities were examined based on quantitative PCR and terminal restriction length polymorphisms of the nitrous oxide reductase (*nosZ*) and 16S rRNA genes. In the stream channel, hydrology had a substantial impact on denitrification rates, likely by significantly lowering water potential in sediments. Clear patterns in denitrification rates were observed among pre-drying, dry, and post-drying dates; however, a less clear scenario was apparent when analyzing bacterial community structure suggesting that denitrifier community structure and denitrification rate were not strongly coupled. This implies that the nature of the response to short-term hydrologic changes was physiological rather than increases in abundance of denitrifiers or changes in composition of the denitrifier community. Flooding of riparian bench soils had a short-term, transient effect on denitrification rate. Our results imply that brief flooding of riparian zones is unlikely to contribute substantially to removal of nitrate (NO_3_^-^) and that seasonal drying of stream channels has a negative impact on NO_3_^-^ removal, particularly because of the time lag required for denitrification to rebound. This time lag is presumably attributable to the time required for the denitrifiers to respond physiologically rather than a change in abundance or community composition.

## Introduction

Humans have drastically altered the global nitrogen (N) cycle through increases in concentrations of fixed N produced for use as fertilizer, yet this is a highly inefficient process as less than 15% of this fixed N serves as a nutritional source for people [1]. Much of the fixed N that is lost enters aquatic environments wherein it can contribute to eutrophication, hypoxia, and violation of drinking water standards. Some microorganisms can ameliorate N pollution; denitrifying bacteria (hereafter, denitrifiers) reduce nitrate (NO_3_^-^) to nitrous oxide (N_2_O) or di-nitrogen gas (N_2_) under anaerobic conditions, thereby removing fixed N from aquatic environments.

Not all parts of the landscape are equal in their ability to support denitrification; headwater streams are able to remove nitrogen via denitrification more efficiently than higher order lotic ecosystems [2], [3]. In addition, the riparian zones of these abundant components of the landscape (headwater streams constitute 90% of stream length in a river system, 4) are the aquatic-terrestrial interface and potentially represent “hot spots” of denitrification [5], [6].

Environmental conditions that potentially drive denitrification meet the energetic and resource needs of denitrifiers and induce this metabolic process; most denitrifiers are facultatively anaerobic heterotrophs [7]. Environmental drivers of denitrification include concentrations of NO_3_^-^, amounts and types of organic carbon, oxygen concentrations, temperature, pH, and occurrence of specific enzyme inhibitors [8]. In streams, NO_3_^-^ concentration is widely reported as the major driver of denitrification (e.g., [9], [10], [11], [12], [13]). Mulholland et al. [3], in a study across 49 streams of varying land use (native vegetation, agricultural, suburban/urban), found that NO_3_^-^ concentrations and hydrology were the most important variables positively affecting N removal by denitrification. Agriculturally impacted streams in the central U.S. typically have high NO_3_^-^ concentrations and high denitrification rates [14], [15].

Denitrification in riparian zones is somewhat less well studied than in other terrestrial environments and in streams (but see [16 and 17] and the review by Martin et al. [18]). In addition, most studies of streams have focused on those with permanent flow (e.g., [15], [19], [20], [21], [22], [23]) while few studies have examined denitrification in ephemeral streams during times of continuous water flow [14]. Thus, it is largely unknown how varying hydrologic regimes in agriculturally impacted streams and riparian buffer zones affect the denitrifying community and denitrification rates. Prior studies demonstrate that inundation of floodplains enhances denitrification in agriculturally impacted watersheds; such inundations occur frequently in tile-drain fed streams of the agricultural midwest [24]. Moreover, restoration practices that promote riparian inundation can enhance floodplain denitrification and reduce N loads [25].

Varied hydrologic and moisture regimes, such as drying and flooding, can alter nitrogen concentrations, ammonia diffusion, and oxygen concentrations [26], [27]. Water regime fluctuations can directly control duration of oxic and anoxic phases in soil, consequently affecting denitrification [28]. Floods in riparian zones can result in pulses of denitrification and the magnitude of this response varies with flood duration [29]. Soil moisture also impacts responses of denitrification to oxygen concentration [30] and the pulse of denitrification post-flood can be sustained by addition of organic compounds [31].

Many prior studies on denitrification have measured biogeochemical processes but have not considered the underlying bacterial community responsible for this process (but see, for example, [14], [32]) Varying moisture content and altered redox potential of seasonally flooded soil and sediment may alter both the community composition (structure) and function of the denitrifier community. In this study, we examined the effect of hydrologic regime on bacterial community composition and denitrification rates in agriculturally impacted streams. The inter-connection between bacterial community structure and function was also examined, as several studies suggest a relationship between rate of denitrification and denitrifier community composition [20], [33]. Exploration of such relationships is important because variation in denitrification potential may be related to properties of the denitrifier community [14], [34], [35].

As environmental conditions become favorable to denitrification (due to flooding, in our case) increased N removal by denitrification may occur because of: 1) increases in the rate of denitrification per cell (in which case the community structure would not change, a physiological response) and/or 2) changes in structure of the denitrifier community (a genetic response). For the latter possibility, the manifestation of these changes could be increases in number of denitrifiers (in this study, based on abundance of the nitrous oxide reductase gene, *nosZ*) or changes in composition of the denitrifier community (in this study, based on terminal restriction fragment length polymorphisms [T-RFLP] of *nosZ*). We predict that the physiological response (increased denitrification rate per cell) will immediately follow flooding whereas changes in the structure of the denitrifier community will lag behind.

Two agriculturally impacted streams in the Sugar Creek watershed in Indiana, USA were used as study sites and sampled periodically. The streams exhibit different hydrologic regimes; one stream is seasonally ephemeral because of its source (tile drainage), whereas the other stream has permanent flow throughout the year. Previous research on these streams, at times when both had flowing water, revealed significantly different denitrifier community composition and denitrification rates between streams [14]. Additionally, a simulated flooding experiment was performed on the riparian benches of the ephemeral stream during a dry period to study the short term effect of flooding. Collectively, this design allowed us to examine more than just in-channel processes and provided two distinct mechanisms by which hydrology was manipulated. Natural hydrologic changes (drying and wetting) were hypothesized to disrupt microbial community structure and/or function resulting in variable denitrification rates. These differences were predicted to be more pronounced in the smaller stream, which experiences complete loss of flow, compared to the larger stream that maintained flow throughout the summer. We tested these hypotheses on seasonal/monthly time scales, which may provide insufficient resolution to see short-term changes. Thus we conducted an experimental manipulation (flooding experiment) to investigate short-term changes in bacterial community structure and denitrification following inundation of riparian soils.

## Materials and Methods

### Study Site and Experimental Design

This study did not involve human or animal subjects. Water, sediment and soil samples were collected from the field with permission from private landowners (for site locations, contact Todd Royer of Indiana University). Samples were collected from Sugar Creek and Leary Weber Ditch (LWD, a Sugar Creek tributary) in Indiana, USA. These streams are agriculturally impacted: 75% and 87% of the Sugar Creek and LWD watersheds, respectively, are corn and soybean fields [36], [37]. The streams were selected based on their differing hydrologic regimes and use in prior studies [14], [38]. Throughout the year, Sugar Creek maintains a permanent flow, whereas LWD typically experiences a dry period with little to no flow during August and September, when tile drains that serve as its water source stop flowing. In addition to differences in hydrology, LWD has riparian benches that experience short periods of flooding during high precipitation events that may alter the denitrifier community and rate of denitrification. The riparian soil of LWD is categorized as Crosby-Brookston, which typically corresponds to very poorly drained clay or silt loam within the top 30 cm of the soil profile [39]. The stream sediments are roughly 80% 1.0 mm and smaller (predominantly coarse and fine sand) in LWD and roughly 70% <1.00 mm in Sugar Creek (Leff, unpublished data).

This study was conducted in two parts. The first phase of the study, described in this paragraph, involved periodic collection of samples from the stream channel over the course of the annual hydrologic cycle. To examine responses to seasonal changes in hydrology, LWD and Sugar Creek were sampled in April, May, September, November, and December 2011. Sediment samples were collected with a plastic corer (6 cm wide × 4 cm deep) from five transects approximately 25 meters apart, along a 100 meter reach of the stream. Simultaneously, at times and locations when standing water was present, water physicochemical variables were measured and samples were collected for water nutrient analysis as described below.

In addition to collection of samples from the stream channel, soil samples were collected with a 2.5 cm wide by 10 cm long corer from riparian benches associated with the 5 transects in LWD. Sugar Creek does not have such benches so soil samples were not collected from that stream. A total of thirty cores were collected; ten cores were used for nutrient analysis, ten for molecular analysis, and ten for denitrification assays.

In the second phase of the study, a flooding experiment was performed at the riparian benches (the floodplain, outside the stream channel) of LWD using flooding enclosures. The enclosures were PVC pipes 60 cm in length by 15 cm in diameter that were pounded approximately 25 cm into the soil of the riparian benches. Enclosures were initially deployed in the summer of 2010 and the flooding experiment was conducted in July 2011 to allow time for the communities in and around the enclosures to recover. Of the six enclosures placed at each bench, three were used as controls and three were flooded with water from the stream to a water depth of 20 cm. Enclosures were flooded for a period of three days, and water removed on the third day via manual suction until all standing water was removed. The third day was considered time zero.At each sampling time point, 2 cm soil cores from within each of the six enclosures were collected for nutrient analysis, molecular analysis, and denitrification assays as described below. Soil samples were collected pre-flood, and 0, 24, and 72 hours after the water was removed.

### Physicochemical Variables

In the field, temperature, pH, dissolved oxygen, and conductivity were measured using a Hach rugged field kit (Hach Company, Loveland, CO), and turbidity was measured using a Hach 2100P turbidimeter. Velocity was measured using a Hach Portable Water Flow meter Model 201 and discharge was calculated using stream width, depth, and water velocity. In the lab, water samples for NO_3_^-^ and soluble reactive phosphorus were filtered through a 0.45 µm filters followed by a 0.22 µm anodisc filter (GE Healthcare Life Sciences, Piscataway, NJ) and analyzed with a Quikchem 800+ FLA (Lachat Instruments, Hach Company). Water samples for dissolved organic carbon (DOC) were filtered through a 0.45 µm filters, a 0.22 µm filter, acidified, and measured using a TOC5000 analyzer (Shimadzu Corporation, Columbia, MD). For sediment and soil samples, percent moisture and organic matter was determined by drying samples at 105°C for 24 h and then combusting at 500°C for 6 hours. Additionally for soil samples, NO_3_^-^ concentrations were measured using KCl extraction; after incubation in 2 M KCl for 24 hours (with shaking) samples were filtered and analyzed as above.

### Biological Variables

Sediment denitrification assays were performed using the chloramphenicol-amended acetylene inhibition method [15], and soil denitrification assays were performed via the static core method [40]. Below, the results of these assays are referred to as denitrification rates in the interest of simplicity. For the former, samples were placed in glass bottles with an air-tight cap and septum and stream water amended with 2 mM chloramphenicol was added before bottles were flushed with helium to ensure an anoxic environment. 15 mL of acetylene was added, and bottles were shaken vigorously. Bottles were incubated at the approximate temperature of the stream, and a headspace sample was collected after the first fifteen minutes, and each hour thereafter for a total of 4 hours and 5 sampling points. After each sampling point, the headspace was replenished with a 10% acetylene/90% helium gas mixture. Headspace gas samples were stored in evacuated gas vials until analyzed on a Shimadzu GC-2014 gas chromatograph (Shimadzu Corporation, Columbia, MD).

Soil denitrification assays were performed via the static core method; samples were collected with 2 cm diameter soil probes and then incubated in acrylic tubes (with septa) [40]. 5 mL of acetylene was added to the tubes, which were then incubated for eight hours at room temperature. Headspace samples were collected every two hours for a total five sampling points. After each sampling point, the headspace was replenished with a 10% acetylene/90% air mixture.

To determine bacterial abundance, sediment sub-samples were weighed and preserved in a 4% paraformaldehyde in phosphate buffered saline. Epifluorescence microscopy was used to determine total bacterial abundance after staining with 4′6-diamidino-2-phenylindole (DAPI) [41].

DNA was extracted from soil and sediment samples using the MoBio Powersoil DNA Isolation Kit (MoBio, Carlsbad, CA); methods used are as described in Baxter et al. [14]. For examination of bacterial community profiles via terminal-restriction fragment length polymorphism (T-RFLP), 16S rRNA genes were amplified using primer sequences described in Blackwood et al. [42]. Primer sequences are provided in Table S1.25 µL reaction mixtures consisted of 2 µL of template DNA, 12.5 µl of water, 0.5 µL of both forward and reverse primers, and 12.5 µL of GoTaq Pre-Mixed Green Master Mix (Promega Corporation, Madison, WI). PCR amplification was carried out using a PTC-200 DNA Engine Cycler (Bio-Rad Laboratories, Hercules, CA) with the following temperature profile: 94°C for 3 minutes, 35 cycles of 94°C (30 s), 57°C (30 s), and 72°C (1 min) with a final extension of 72°C for 7 min. PCR was also used to generate 700 bp *nosZ* fragments from each sample for T-RFLP; *nosZ* has been used in previous studies to examine denitrifier community composition [33]. Primers used were from Rich et al. [33]. 25 µL reaction mixtures consisted of 2 µL of template DNA, 12.5 µL of water,0.5 µL of forward primer, 0.5 µL of reverse primer, and 12.5 µL of GoTaq Pre-Mixed Green Master Mix (Promega Corporation). PCR amplifications were carried out using a PTC-200 DNA Engine Cycler (Bio-Rad Laboratories), with the following temperature profile: 94°C for 3 minutes, 35 cycles of 94°C (45 s), 55°C (1 min), and 72°C (2 min), with a final extension at 72°C (7 min). For all samples, five PCR reactions were pooled and purified using a Wizard SV Gel and PCR Clean-Up System (Promega Corporation). PCR product sizes were confirmed using gel electrophoresis.

Fluorescently labeled 16S rRNA and *nosZ* gene PCR products were digested using 2 U of restriction endonuclease HaeIII and RsaI, respectively, at 37°C as in Baxter et al. [14] and Feinstein et al. [43]. Post digestion, samples were purified using the Wizard SV Gel and PCR Cleanup System (Promega Corporation) and were sent to The Ohio State University Plant Microbe Genomics Facility for T-RFLP analysis on a 3730 DNA Analyzer (Applied Biosystems, Life Technologies Corporation, Carlsbad, CA).

Quantitative PCR was used to determine the abundance of 16S rRNA genes. Primer sequences used were from Fierer et al. [44]. 25 µL reaction mixtures consisted of 2 µL of template DNA, 10.5 µL of water, 0.25 µL of both forward and reverse primers, and 12.5 µL of SYBR Green PCR Master Mix (Applied Biosystems). The quantitative PCR temperature profile on a Stratagene MX3005P Real-Time PCR System (Agilent Technologies, Santa Clara, CA) for DNA amplification was 94°C (10 min), 40 cycles of 94°C (30 s), 57°C (1 min), and 72°C (30 s). Beginning at 55°C, a dissociation curve was produced from forty 30 second cycles that increased 1°C per cycle.

Denitrifier abundance was based on the quantification of *nosZ* genes determined via quantitative PCR, and primer sequences used are from Henry et al. [45]. 25 µL reaction mixtures consisted of 2 µL of template DNA, 10.5 µL of water, 0.25 µL of both forward and reverse primers, and 12.5 µL of SYBR Green PCR Master Mix (Applied Biosystems). The quantitative PCR temperature profile on a Stratagene MX3005P Real-Time PCR System (Agilent Technologies) for DNA amplification was 95°C (10 min), 40 cycles of 94°C (45 s), 57°C (1 min), 72°C (2 min), and 80°C (15 s). The dissociation curve was produced from forty 30 second cycles increasing 1°C per cycle, starting at 55°C.

### Statistical Analyses

For the temporal study, two-way ANOVA was used to test for significant differences between sites and dates. When a significant main effect or interaction was found using the two-way ANOVA, Tukey's *post hoc* multiple comparison test was used to determine which means differed. For the simulated flood experiment, mixed model ANOVA was used to assess differences between riparian bench soil cores collected at different time points before and after the flooding treatment.

Analysis of T-RFLP results was based on Blackwood et al. [46] and the references cited below. T-RFLP results were analyzed using band-matching analysis in GelComparII (Applied Maths Inc., Austin, TX). Specifically, size, height and area data for each peak were imported into GelComparII and peaks with sizes less than 50 or greater than 600 bp were excluded. In addition, peaks which contributed less than 0.5% of the total area were also excluded (as in [43]). Then, redundancy analyses (RDA) were performed in version 2.11.1 of R [47] to examine which factors contributed significantly to variation between [14], [32], [33], [42], [48]. Overall, this technique analyzes differences in relative peak heights as well as peak presence or absence between profiles (by including the positions of peaks in profiles in the analysis).

## Results

### Temporal Study

Physical and chemical variables are summarized in Table 1; distinct patterns of temporal change were observed along with differences between the two study streams. For example, LWD had significantly lower discharge (average = 28 L/s) than Sugar Creek (average = 226 L/s). On average, turbidity was twice as high in LWD compared to Sugar Creek (8.0 and 4.3 NTU, respectively) while benthic organic matter content was three times higher in LWD than in Sugar Creek (3.5 and 1.3%, respectively).

**Table 1:** Mean (±1 SE) percent benthic organic matter (% OM); other variables are from water samples: temperature (temp), pH, turbidity, dissolved oxygen (DO), specific conductivity, discharge, and soluble reactive phosphorus (SRP) by stream for each date.

Date	Stream	% OM	Temp (°C)	pH	Turbidity (NTU)	DO (mg/L)	Specific Conductivity (µS/cm @ 25°C)	Discharge (L/s)	SRP (µg/L)
**April**	**LWD**	3.30%	11.08	7.64	4.89	10.78	637.38	81.30	17.96
		(±0.54)	(±0.60)	(±0.40)	(±0.31)	(±0.71)	(±0.65)	(±0.42)	(±0.70)
	**Sugar Cr**	1.26%	10.80	8.26	3.17	9.06	366.40	374.60	13.21
		(±0.13)	(±0.14)	(±0.24)	(±0.60)	(±0.18)	(±6.15)	(±0.13)	(±0.48)
**May**	**LWD**	4.20%	11.30	7.60	7.04	10.74	729.60	32.80	8.25
		(±0.51)	(±0.90)	(±1.21)	(±1.03)	(±0.60)	(±6.71)	(±0.84)	(±0.22)
	**Sugar Cr**	1.30%	11.66	7.73	8.81	10.28	737.80	286.40	22.64
		(±0.06)	(±0.60)	(±0.10)	(±0.10)	(±0.70)	(±1.02)	(±0.90)	(±0.32)
**Sept**	**LWD**	nd	nd	nd	nd	nd	nd	0.00(±0.00)	nd
									
	**Sugar Cr**	1.37%	15.00	7.42	3.82	9.35	546.2	62.8	14.51
		(±0.10)	(±0.40)	(±0.63)	(±1.25)	(±0.94)	(±2.71)	(±0.17)	(±0.17)
**Nov**	**LWD**	3.40%	14.06	7.40	10.91	10.45	756.6	16.20	9.12
		(±0.29)	(±0.60)	(±0.10)	(±0.16)	(±1.11)	(±2.01)	(±0.70)	(±0.39)
	**Sugar Cr**	1.20%	15.02	7.13	2.62	8.15	444.8	214.70	16.98
		(±0.04)	(±0.44)	(±0.30)	(±0.36)	(±1.86)	(±3.71)	(±0.60)	(±0.20)
**Dec**	**LWD**	3.41%	10.8	7.35	9.30	10.54	763.6	12.3	9.36
		(±0.29)	(±0.14)	(±0.34)	(±0.19)	(±0.96)	(±1.16)	(±0.72)	(±0.22)
	**Sugar Cr**	1.65%	8.95	7.15	2.75	8.86	482.8	194.40	13.01
		(±0.11)	(±0.84)	(±0.40)	(±0.11)	(±1.18)	(±4.67)	(±0.77)	(±0.20)

There was no standing water at the LWD site in Sept.

**Figure 1:**
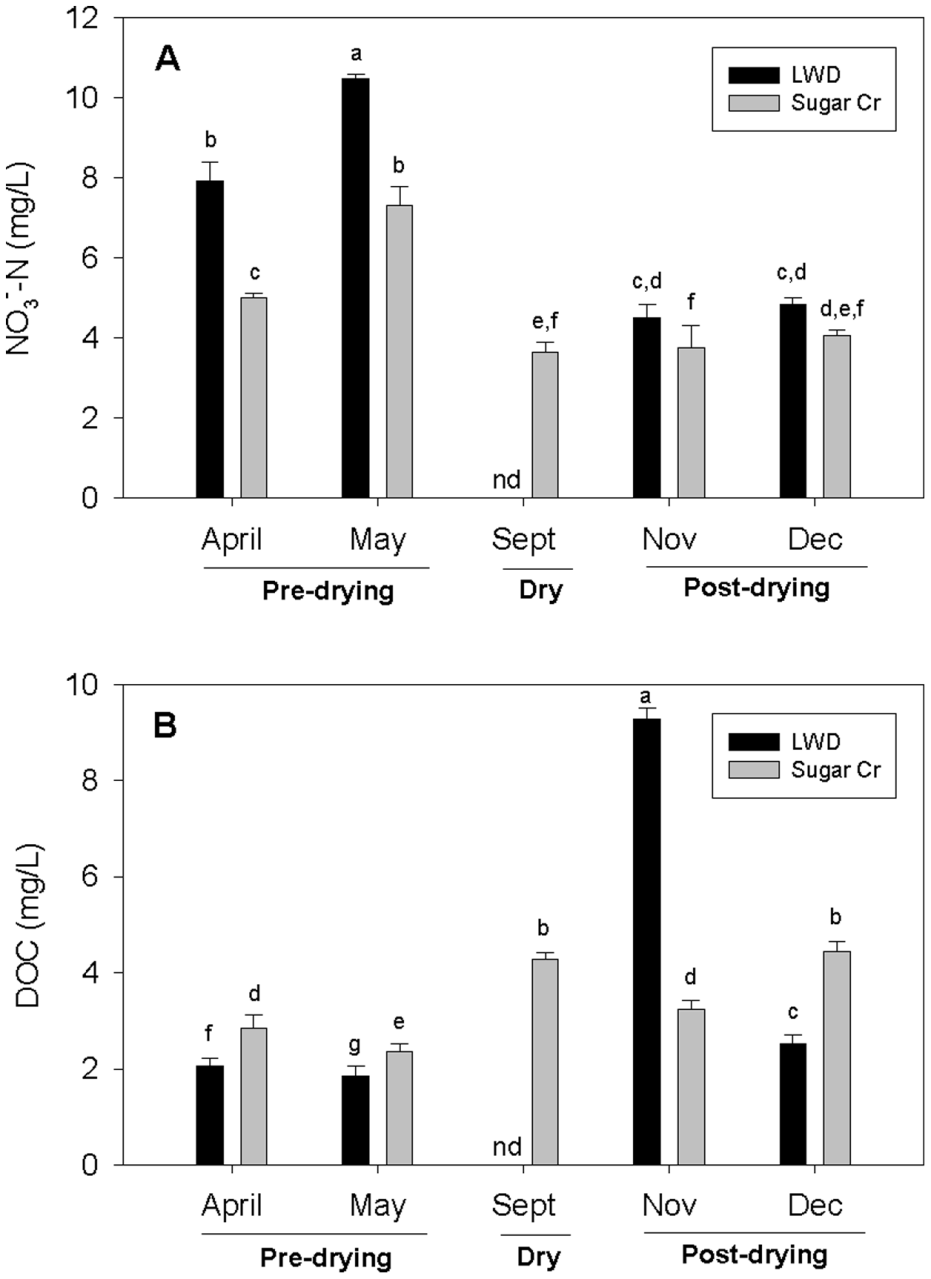
Nitrate and dissolved organic carbon concentrations in study streams. (A) Mean NO_3_^-^ concentrations (mg N/L) by stream for each date. (B) Mean dissolved organic carbon (DOC) concentrations (mg/L) by stream for each date. Pre-drying indicates LWD had flow; dry, no flow; post-drying, flow returned. Letters indicate significant differences (p<0.05) based on Tukey's *post hoc* multiple comparisons (note the highest value will always be ‘a’). Error bars indicate +1 SE.

Nitrate concentrations differed significantly between the two streams (p<0.05), as well as among dates (p<0.05); the stream by date interaction was also significant (p<0.05). Concentrations of NO_3_^-^ were generally higher in spring (April and May) compared to other dates in both LWD and Sugar Creek (p<0.05, Figure 1A). Subsequently, NO_3_^-^ concentrations dropped roughly two-fold in both streams in November and December to less than 5 mg N/L in LWD and less than 4 mg N/L in Sugar Creek. NO_3_^-^ concentrations tended to be higher in LWD than Sugar Creek; average NO_3_^-^ concentrations across all dates in LWD and Sugar Creek were 6.94 mg N/L and 4.76 mg N/L, respectively.

Dissolved organic carbon (DOC) concentrations differed between the two streams (p<0.05), as well as among dates (p<0.05); the stream by date interaction was also significant (p<0.05). DOC concentrations were generally higher in the fall and winter (November and December) in both streams (p<0.05, Figure 1B) and DOC concentration in LWD in November was 2–3 times higher than other samples (p<0.05). Lowest DOC concentrations were in April and May (p<0.05). LWD averaged higher DOC concentrations (across all dates) than Sugar Creek (4.11 mg/L and 3.29 mg/L, respectively).

**Figure 2:**
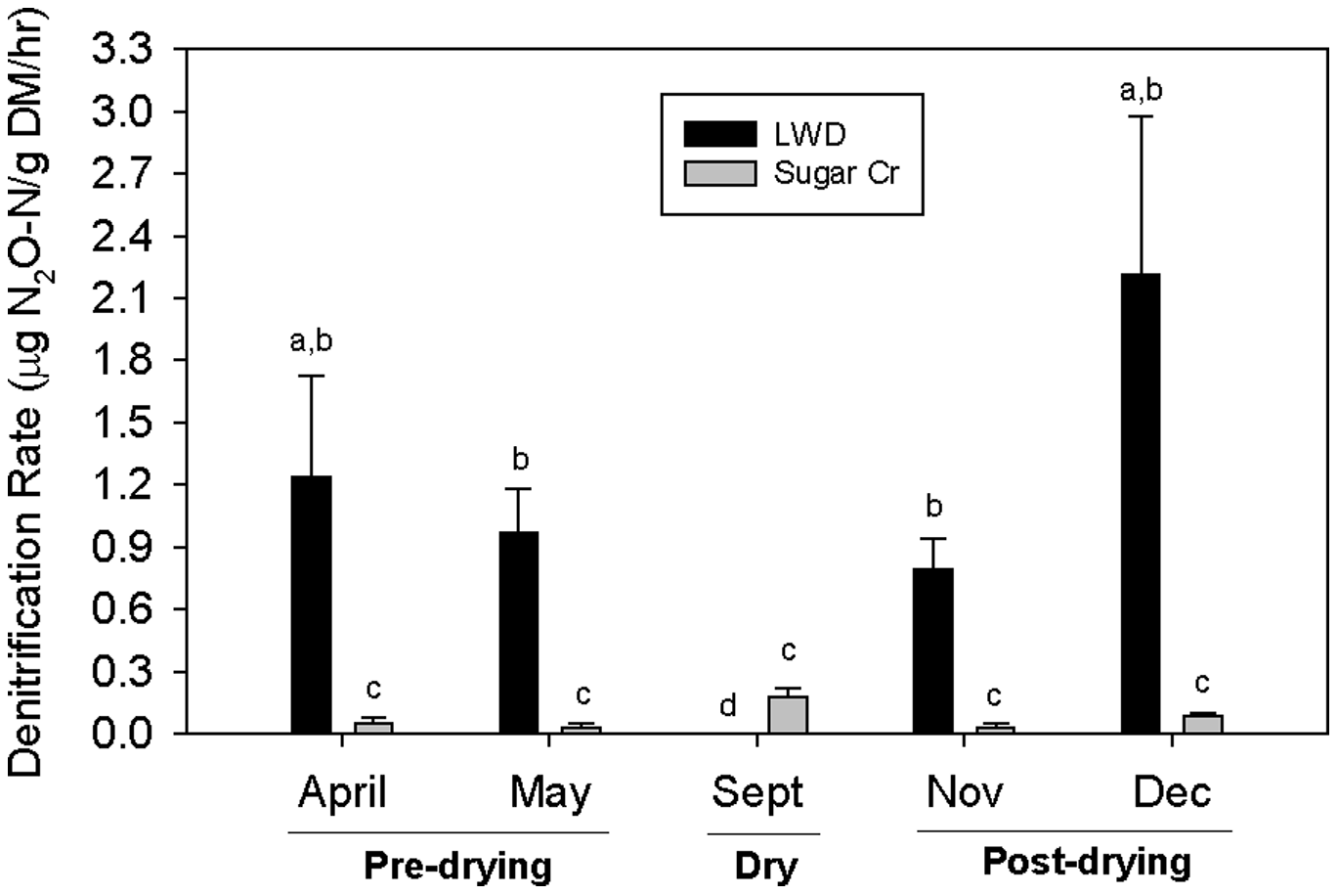
Mean denitrification rate by stream for each date. Pre-drying indicates LWD had flow; dry, no flow; post-drying, flow returned. Letters indicate significant differences (p<0.05) based on Tukey's *post hoc* multiple comparisons. Error bars indicate +1 SE.

Denitrification rates significantly varied between the two streams (p<0.05), as well as among dates (p<0.05); the stream by date interaction was also significant (p<0.0005). LWD denitrification rates were consistently 20–30 times higher than those in Sugar Creek in April, May, November, and December (p<0.05, Figure 2). However, the denitrification rate in LWD in September (when the stream was dry) was significantly lower than any other measured rate in either stream among dates (p<0.05).

**Figure 3:**
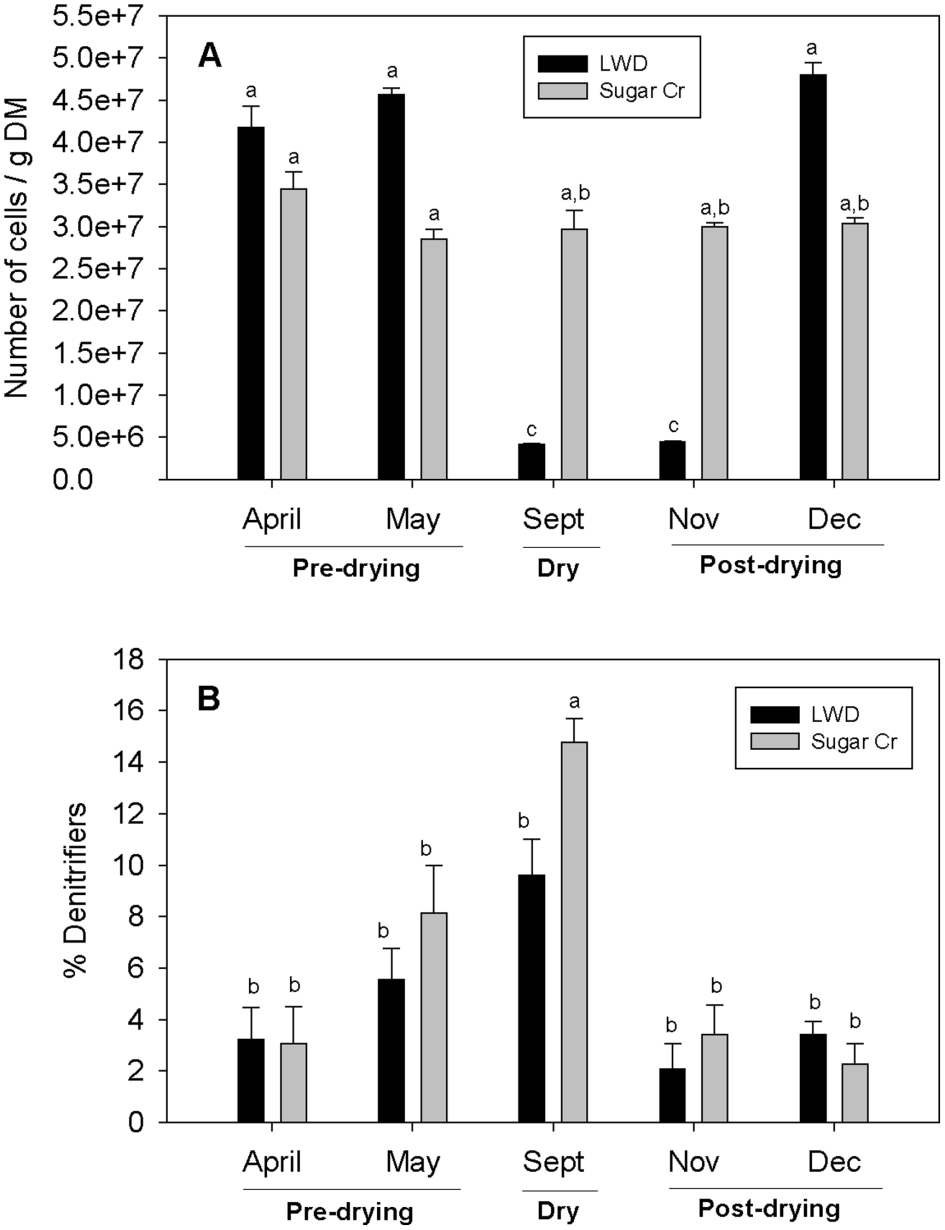
Bacterial abundance in study streams. (A) Mean total bacterial abundance (based on DAPI counts) by stream for each date. (B) Mean denitrifier abundance (ratio of *nosZ*: 16S rRNA copy number) by stream for each date. Pre-drying indicates LWD had flow; dry, no flow; post-drying, flow returned. Letters indicate significant differences (p<0.05) based on Tukey's *post hoc* multiple comparisons. Error bars indicate +1 SE.

Total bacterial abundance (based on DAPI counts) differed between the two streams (p<0.05), and among dates (p<0.05); the stream by date interaction was also significant (p<0.05). Bacterial abundance was similar in both LWD and Sugar Creek in April and May; these dates had the highest bacterial abundance across all dates (p<0.05, Figure 3A). In Sugar Creek, after spring (April and May) bacterial abundances declined slighting (abundances in September, November, and December were similar). LWD bacterial abundance decreased significantly in September and November, when flow was absent or had just returned; bacterial abundance for these dates was significantly lower than at any other time (p<0.05).

Relative denitrifier abundance (ratio of *nosZ*:16S rRNA copy number) did not differ between streams (p>0.05), but was significantly different among dates (p<0.05); the stream by date interaction was also significant (p<0.05). The highest percentage of denitrifiers was in September in Sugar Creek (p<0.05, Figure 3); this site had a denitrifier abundance two to twenty-five times higher than any other site across dates. In May and November in Sugar Creek and LWD, and September in LWD, relative denitrifier abundances were similar to each other; abundances on these dates were four to ten times higher than April and November denitrifier abundances (p<0.05).

**Figure 4:**
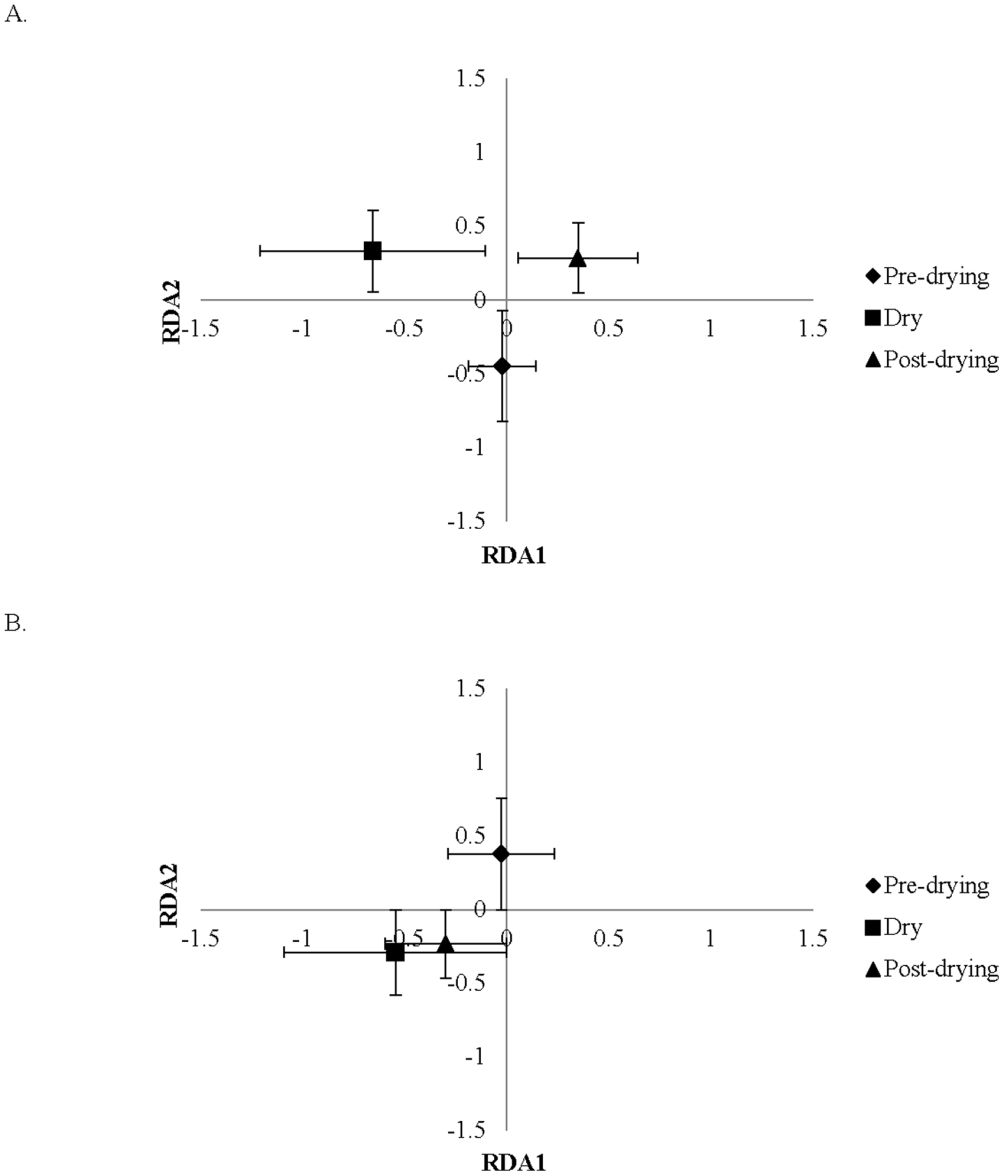
Statistical analysis of bacterial community structure in study streams. (A)Redundancy analysis of LWD 16S rRNA T-RFLP profiles by hydrologic conditions. Stream hydrologic condition explained 40.21% of community variation. (B) Redundancy analysis of LWD *nosZ* T-RFLP profiles by hydrologic conditions. Stream hydrologic condition explained 45.30% of denitrifier community variation.

16S rRNA bacterial community T-RFLP profiles based on RDA were not significantly different between streams (p>0.05). To examine the responses to seasonal drying, LWD samples were then analyzed separately and differed significantly among pre-drying, dry, and post-dry sample dates (p<0.05). Hydrologic condition (pre-drying, dry, post-dry) explained 40.2% of the variation in 16S rRNA bacterial communities (p<0.05, Figure 4A).

*nosZ* bacterial community profiles followed a similar pattern; community profiles were not significantly different between streams (p = 0.25). When results from LWD were analyzed separately there were significant differences among pre-drying, dry, and post-dry sample dates (p<0.05, Figure 4B). Hydrologic conditions (pre-drying, dry, post-drying) explained 45.3% of variation in denitrifier communities.

### Flood Experiment

**Figure 5:**
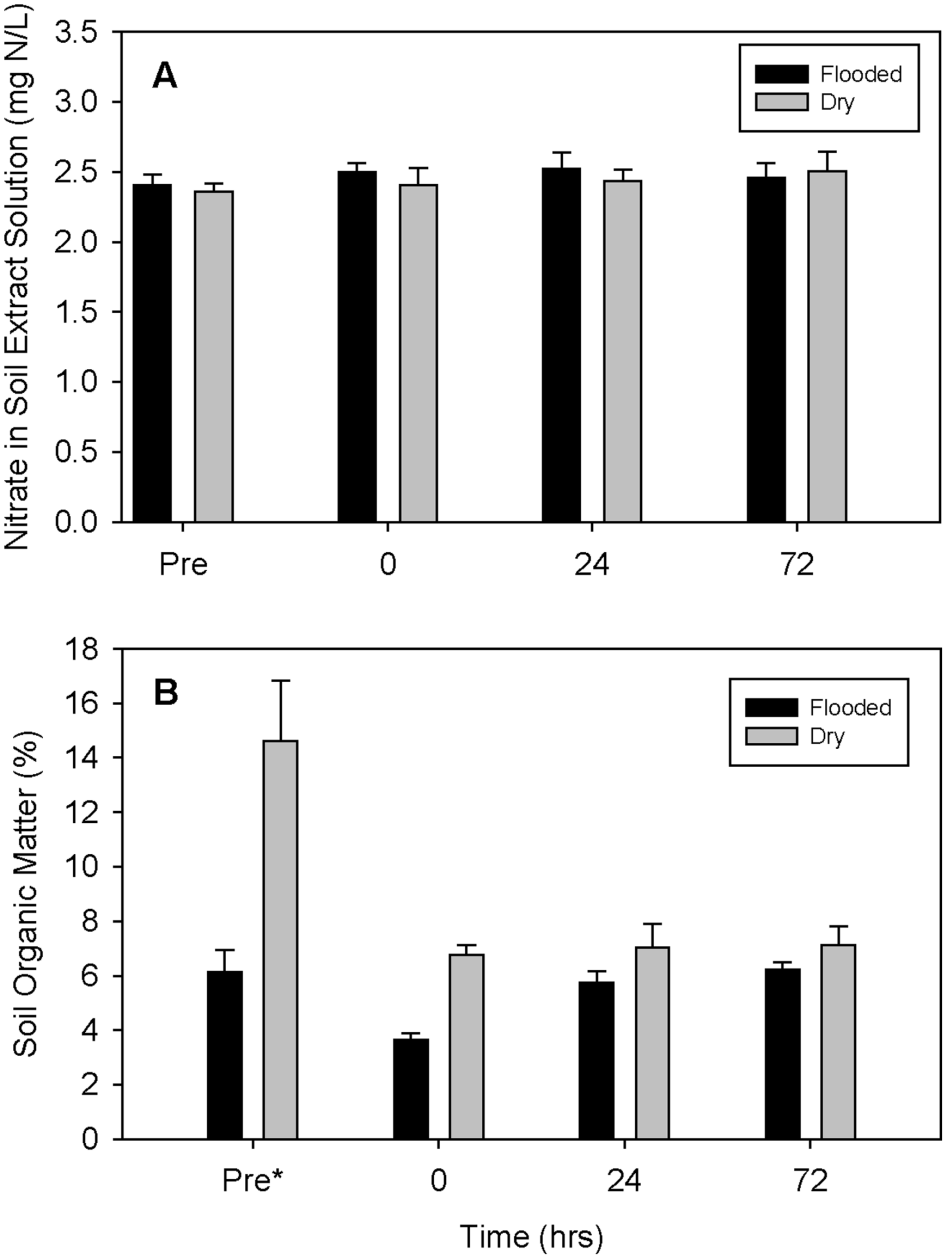
Nitrate and organic matter content of soils sampled. (A) Mean soil NO_3_^-^ concentrations (mg N/L) by treatment over time (per volume of extract). No significant differences were detected. (B) Mean percent organic matter by treatment over time. The pre-flood collection time point was significantly different from time zero, 24 hours and 72 hours post-flood (*p<0.05). Error bars indicate +1 SE.

Soil NO_3_^-^ concentrations (measured via KCl extraction, expressed per volume of extract) were not significantly different among bench sites (p>0.05), enclosures (p>0.05), treatments p>0.05) or collection time points (p>0.05); the treatment by time interaction was also not significant (p>0.05, Figure 5A). Soil organic matter content was also measured from within the flooding enclosures; the percentage of organic matter differed among collection time points (p<0.05) and treatments (p<0.05); the treatment by time interaction was also significant (p<0.05). In contrast, there were no significant differences among bench sites (p<0.05, Figure 5B) or enclosures within a bench (p>0.05). Although the control enclosures appeared to have consistently higher organic matter content than flooded enclosures, Tukey's *post hoc* multiple comparisons revealed no significant differences.

**Figure 6:**
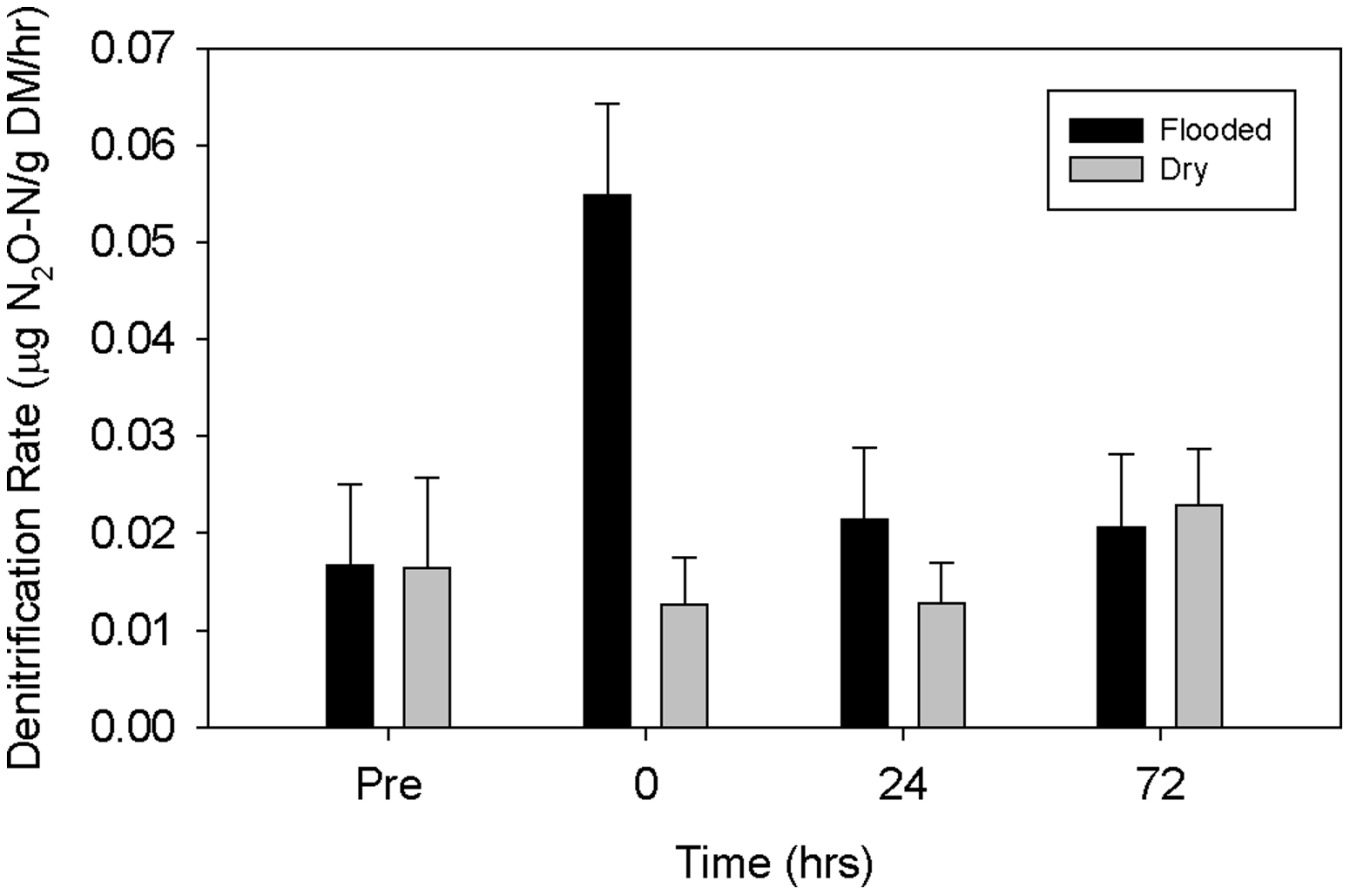
Mean denitrification rate by treatment over time. No significant differences were detected. Error bars indicate +1 SE.

Denitrification rates significantly differed among bench sites (p<0.05); the treatment by time interaction was also significant (p<0.05; Figure 6). No significant differences were detected among enclosures (p>0.05), collection time points (p>0.05) or treatments (p>0.05). Although the treatment by time interaction was significant (p<0.05), suggesting that differences among treatment depended on the time point of sample collection, Tukey's *post hoc* multiple comparison revealed no significant differences in denitrification rate between flooded and control enclosures.

**Figure 7:**
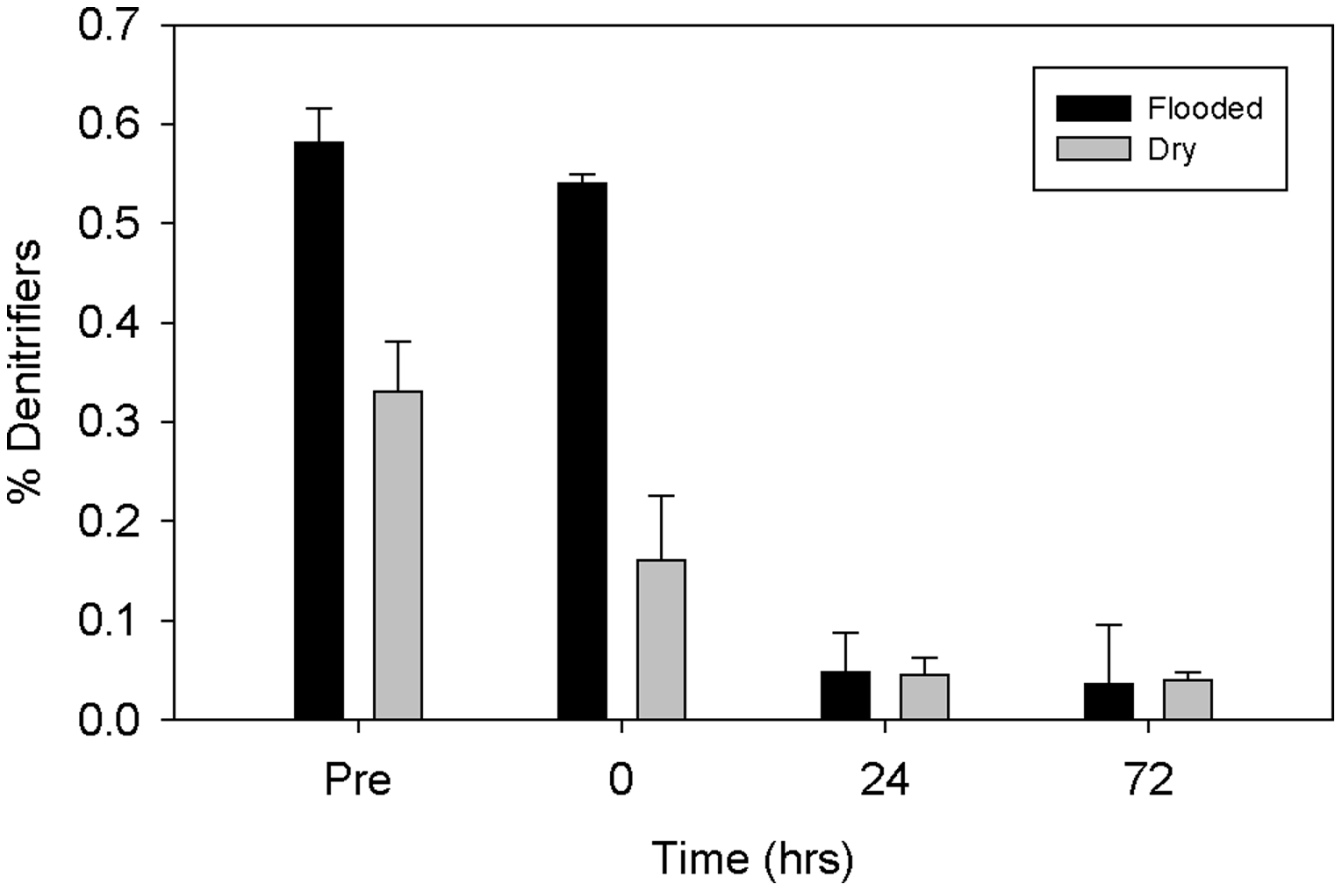
Mean denitrifier abundance (ratio of *nosZ*: 16S rRNA copy number) by treatment over time. Error bars indicate +1 SE.

Relative denitrifier abundance (ratio of *nosZ*:16S rRNA copy numbers) was significantly different between bench sites (p<0.05); the treatment by time interaction was also significant (p<0.05) (note: total bacterial numbers were not determined for bench samples). No significant differences in denitrifier abundance were detected among enclosures (p>0.05), collection time points (p>0.05), or between treatments (p>0.05). Although the treatment by time interaction was significant (p<0.05), Tukey's *post hoc* multiple comparison revealed no significant differences between flooded and control enclosures over time (Figure 7).

**Figure 8:**
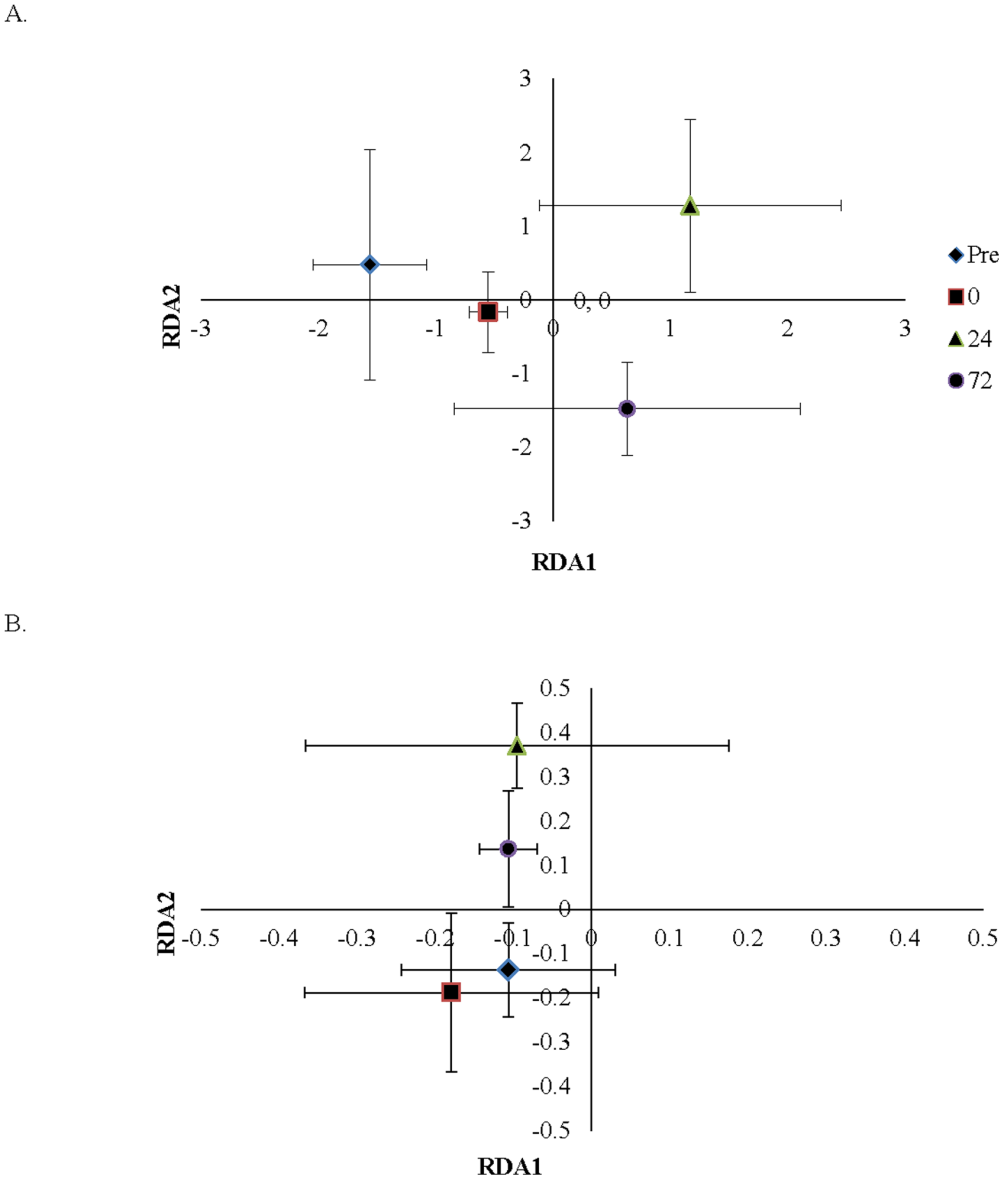
Statistical analysis of bacterial community structure in soils sampled. (A) Redundancy analysis of 16S rRNA T-RFLP profiles by treatment over time. Collection time point explained 6.66% of community variation. (B) Redundancy analysis of *nosZ* T-RFLP profiles by treatment over time. Collection time point explained 3.61% of community variation

Redundancy analysis of 16S rRNA T-RFLP profiles revealed no statistically significant differences among treatments, or bench site; while differences among time points were significant (p<0.05). However, collection time point explained 6.7% of community variation (Figure 8A). Likewise, *nosZ* bacterial community T-RFLP profiles were significantly different among collection time points (p<0.05), whereas no significant differences were detected among bench sites (p>0.05) or between treatments (p>0.05). Yet, collection time point only explained 3.6% of *nosZ* bacterial community variation (Figure 8B).

## Discussion

Moisture content of soil, which is directly controlled by hydrology, plays a critical role in determining effectiveness of denitrification in NO_3_^-^ removal (e.g. [28]). During times when it was flowing, LWD had higher denitrification rates than Sugar Creek and lower discharge. However, when LWD had no flow and was dry (September), denitrification rate dropped below that of Sugar Creek. Consistent with these findings, Pinay et al. [28] determined that soil moisture was the most important factor in predicting rates of denitrification in streams with alternating flooding and drying cycles.

When the dry sampling date at LWD is excluded, denitrification rates in LWD were much higher than in Sugar Creek. Prior studies demonstrate that NO_3_^-^ is among the most potent environmental drivers of denitrification (e.g. [3], [11], [49]) along with temperature and sediment organic matter content [12], [31], [49], [50]. In the present study, LWD had three times higher benthic organic matter than Sugar Creek and higher NO_3_^-^ concentration (averages were 6.94 mg N/L and 4.76 mg N/L respectively). Likewise, in a study of these same streams, Baxter et al. [14] found that LWD had higher rates of denitrification than Sugar Creek in summer. However, no significant differences between streams were observed in fall in that study, in contrast to the present study. In general, denitrification rates we observed are consistent with other studies of agriculturally impacted streams [21], [51], [52] but rates in Sugar Creek were somewhat low, relatively, perhaps because of comparatively lower NO_3_^-^ concentrations (which in absolute terms are high and indicative of high agricultural land use) and higher discharge [11], [12], [53].

At the same time that denitrification rates dropped in LWD with seasonal drying, total bacterial numbers declined perhaps because of elevated bacterial mortality or reduced growth [54], [55]. A lag period was also observed; bacterial cell numbers did not reach pre-drying levels until December, roughly one month following the return of stream flow. To examine the response of denitrifiers, *nosZ* gene abundances were examined because variation in denitrification potential within streams may be related to abundance of denitrification genes ([34], [35]. *nosZ* gene copy numbers revealed that relative abundance of denitrifiers was higher in Sugar Creek than in LWD and that there were no significant differences in LWD between when the stream was flowing and when it had stopped. Some prior studies have not found a relationship between properties of the denitrifier community and denitrification rate (i.e. [33], [56]), while others have suggested that changes in abundance of the *nosZ* gene are a good predictor of N_2_O emissions [57].

Overall bacterial community structure (based on the 16S rRNA gene) and denitrifier community structure (based on the *nosZ* gene) had very similar patterns; T-RFLP profiles of both genes were strongly impacted by seasonal changes. Yet, differences between streams did not account for a significant portion of the variation in bacterial community structure. Likewise, the lack of difference between bacterial community profiles in LWD and Sugar Creek is similar to findings reported by Baxter et al. [14]. This is in contrast to other studies, such as Wilson et al. [58] who determined that flooding of soils induced changes in bacterial community structure.

Another approach to examining hydrological impacts on denitrification is to perform manipulative experiments in the field. Previous research has suggested that riparian zones are favorable areas for denitrification (e.g. [18], [20], [59]) and that flooding of riparian zones can significantly influence environmental conditions needed for denitrification (e.g. [60. 61]). In the present study, there was a significant interaction between flood treatment and time but the increase in denitrification caused by the flood treatment was short-term (less than 24 hr). Although numerous studies have implicated hydrology as an important factor controlling denitrification (e.g. [28, 60. 61, 62, 63]), it remains unclear whether denitrification can immediately occur following the onset of anaerobic conditions. Specifically, Fellows et al. [31] found a lag in denitrification rates of 2–3 days following the beginning of anaerobic conditions.

In the flooding experiment, bacterial and denitrifier community structure exhibited very similar patterns as they did in the temporal study; T-RFLP analysis indicated collection time point was the only measured variable driving community differences. Other studies have found similar results in regard to variations in hydrology effecting bacterial community structure. For example, Song et al. [64] found that in wetlands, hydrologic pulsing (i.e. water level draw down followed by a flooding event) did not have a significant impact on 16S rRNA or denitrifier community structure (using the *nirS* gene).

The temporal study indicates hydrology has a substantial impact on denitrification rates, likely by significantly lowering water potential in sediments. Clear patterns in denitrification rates were observed among pre-drying, dry, and post-drying dates; however, a less clear scenario was apparent when analyzing bacterial community structure suggesting that denitrifier community structure and denitrification rate were uncoupled. This implies that the nature of the short-term response to hydrologic changes was physiological rather than increases in abundance of denitrifiers or changes in the composition of the community. Relative denitrifier abundance and denitrifier community profiles indicated no significant differences between streams, yet denitrification rates in LWD (excluding the dry sampling date) were significantly higher than in Sugar Creek likely because of differences in NO_3_^-^ concentrations, benthic organic matter, and discharge. Manipulating hydrology in the field, rather than collecting samples from the field over the course of natural hydrological changes, resulted in a different view of hydrological impacts. Simulating a flood had a short-term, transient effect on denitrification rate. Our results imply that brief flooding of riparian zones is unlikely to contribute substantially to removal of NO_3_^-^, although extended periods of inundation might be effective (e.g., [65]). We also found that seasonal drying of stream channels had a negative impact on NO_3_^-^ removal, particularly because of the time lag required for denitrification to rebound after flow returned to the channel.

## Supporting Information

Table S1 **Primers used in the study.**
(DOCX)

Data S1 **Bacterial abundance, denitrification rates, and physiochemcial data collected during the study.**
(DOCX)

Data S2 **T-RFLP profiles from temporal study.**
(XLSX)

Data S3 **T-RFLP profiles from flood experiment.**
(XLSX)
