# Supplementary material for: Denitrification in Agriculturally Impacted Streams: Seasonal Changes in Structure and Function of the Bacterial Community
Source: PLoS One. 2014 Aug 29;9(8):e105149. doi: 10.1371/journal.pone.0105149 (PMC4149370; doi:10.1371/journal.pone.0105149)
Supplement: Table S1 — Primers used in the study. (DOCX) [file pone.0105149.s001.docx]

Supplementary Table 1. PCR primers used in this study and references from which sequences were obtained. Primers that were used in mixtures were utilized at equimolar ratios.

| **Target** | **Method** | **Sequence** | **Reference** |
| --- | --- | --- | --- |
| 16S | T-RFLP | F: ACTCCTACGGGAGGCWGC  + ACACCTACGGGTGGCWGC | Blackwood et al. (2005) |
|  |  | R: ACGGGCGGTGTGTACA |  |
|  | qPCR | F: ACTCCTACGGGCAGCAG+  ACACCTACGGGTGGCTGC+ ATTACCGCGGCTGCTGG | Fierer et al. (2005) |
|  |  | R: ATTACCGCGGCTGCTGG |  |
| *nosZ* | T-RFLP | F: CGCTGTTCITCGACAGYCAG | Rich et al. (2003) |
|  |  | R: ATGTGCAKIGCRTGGCAGAA |  |
|  | qPCR | F: ATCCGCTTGTTCACTCGACAGCCAG | Henry et al. (2006) |
|  |  | R: CAGTAGTGCAGTCGGCRTGGCAGAA |  |
