# Supplementary material for: Denitrification in Agriculturally Impacted Streams: Seasonal Changes in Structure and Function of the Bacterial Community
Source: PLoS One. 2014 Aug 29;9(8):e105149. doi: 10.1371/journal.pone.0105149 (PMC4149370; doi:10.1371/journal.pone.0105149)
Supplement: Data S1 — Bacterial abundance, denitrification rates, and physiochemcial data collected during the study. (DOCX) [file pone.0105149.s002.docx]

**Temporal study**

Physicochemical data

|  |  |  |  |  |  |  |  |  |  |  |  |  |
| --- | --- | --- | --- | --- | --- | --- | --- | --- | --- | --- | --- | --- |
| **Date** | **Stream** | **% OM** | **Temp** (^o^C) | **pH** | **Turbidity** (NTU) | **DO**  (mg/L) | **Conductivity** (µS) | **Discharge** (L/s) | **Nitrate** (mg/L) | **SRP**  (µg/L) | **DOC** (mg/L) |  |
| **April** | **LWD** | 3.30% | 11.08 | 7.64 | 4.89 | 10.78 | 637.38 | 81.30 | 7.92 | 17.96 | 2.06 |  |
|  |  | (± 0.54) | (± 0.60) | (± 0.40) | (± 0.31) | (± 0.71) | (± 0.65) | (± 0.42) | (± 0.49) | (± 0.70) | (± 0.20) |  |
|  | **SS** | 1.26% | 10.80 | 8.26 | 3.17 | 9.06 | 366.40 | 374.60 | 5.01 | 13.21 | 2.84 |  |
|  |  | (± 0.13) | (± 0.14) | (± 0.24) | (± 0.60) | (± 0.18) | (± 6.15) | (± 0.13) | (± 0.11) | (± 0.48) | (± 0.30) |  |
| **May** | **LWD** | 4.20% | 11.30 | 7.60 | 7.04 | 10.74 | 729.60 | 32.80 | 10.47 | 8.25 | 1.84 |  |
|  |  | (± 0.51) | (± 0.90) | (± 1.21) | (± 1.03) | (± 0.60) | (± 6.71) | (± 0.84) | (± 0.12) | (± 0.22) | (± 0.20) |  |
|  | **SS** | 1.30% | 11.66 | 7.73 | 8.81 | 10.28 | 737.80 | 286.40 | 7.31 | 22.64 | 2.37 |  |
|  |  | (± 0.06) | (± 0.60) | (± 0.10) | (± 0.10) | (± 0.70) | (± 1.02) | (± 0.90) | (± 0.49) | (± 0.32) | (± 0.21) |  |
| **Sept** | **LWD** | nd | nd | nd | nd | nd | nd | 0 | nd | nd | nd |  |
|  |  |  |  |  |  |  |  |  |  |  |  |  |
|  | **SS** | 1.37% | 15.00 | 7.42 | 3.82 | 9.35 | 546.2 | 62.8 | 3.66 | 14.51 | 4.29 |  |
|  |  | (± 0.10) | (± 0.40) | (± 0.63) | (± 1.25) | (± 0.94) | (± 2.71) | (± 0.17) | (± 0.24) | (± 0.17) | (± 0.10) |  |
| **Nov** | **LWD** | 3.40% | 14.06 | 7.40 | 10.91 | 10.45 | 756.6 | 16.20 | 4.5 | 9.12 | 9.28 |  |
|  |  | (± 0.29) | (± 0.60) | (± 0.10) | (± 0.16) | (± 1.11) | (± 2.01) | (± 0.70) | (± 0.35) | (± 0.39) | (± 0.20) |  |
|  | **SS** | 1.20% | 15.02 | 7.13 | 2.62 | 8.15 | 444.8 | 214.70 | 3.76 | 16.98 | 2.51 |  |
|  |  | (± 0.04) | (± 0.44) | (± 0.30) | (± 0.36) | (± 1.86) | (± 3.71) | (± 0.60) | (± 0.56) | (± 0.20) | (± 0.20) |  |
| **Dec** | **LWD** | 3.41% | 10.8 | 7.35 | 9.30 | 10.54 | 763.6 | 12.3 | 4.85 | 9.36 | 3.25 |  |
|  |  | (± 0.29) | (± 0.14) | (± 0.34) | (± 0.19) | (± 0.96) | (± 1.16) | (± 0.72) | (± 0.17) | (± 0.22) | (± 0.20) |  |
|  | **SS** | 1.65% | 8.95 | 7.15 | 2.75 | 8.86 | 482.8 | 194.40 | 4.05 | 13.01 | 4.44 |  |
|  |  | (± 0.11) | (± 0.84) | (± 0.40) | (± 0.11) | (± 1.18) | (± 4.67) | (± 0.77) | (± 0.15) | (± 0.20) | (± 0.20) |  |

Denitrification Rate

| **LWD** | Rate | SE |
| --- | --- | --- |
| Apr | 1.24138 | 0.485234 |
| May | 0.973029 | 0.209496 |
| Sept | 0.000965 | 0.000423 |
| Nov | 0.7921 | 0.15159 |
| Dec | 2.211759 | 0.763097 |
|  |  |  |
| **SS** | Rate | SE |
| Apr | 0.050344 | 0.029729 |
| May | 0.029106 | 0.020251 |
| Sept | 0.180504 | 0.043107 |
| Nov | 0.032998 | 0.020251 |
| Dec | 0.085998 | 0.012976 |

nosZ gene copy numbers (/g GM and % of 16S)

| LWD | avg | sterr | SS | avg | sterr |
| --- | --- | --- | --- | --- | --- |
| Apr | 1.94E+08 | 1.01E+07 |  | 1.12E+08 | 1.41E+07 |
| May | 2.06E+08 | 1.90E+07 |  | 2.49E+08 | 2.13E+07 |
| Sept | 3.43E+07 | 5.03E+06 |  | 6.27E+07 | 5.77E+06 |
| Nov | 7.78E+07 | 4.52E+06 |  | 2.14E+07 | 1.54E+06 |
| Dec | 5.76E+07 | 2.29E+06 |  | 2.05E+07 | 3.07E+06 |

|  | LWD | | SS | |
| --- | --- | --- | --- | --- |
|  | avg | sterr | avg | sterr |
| Apr | 3.23% | 1.23% | 3.05% | 1.46% |
| May | 5.55% | 1.22% | 8.15% | 1.82% |
| Sept | 9.60% | 1.41% | 14.77% | 0.93% |
| Nov | 2.08% | 0.97% | 3.43% | 1.13% |
| Dec | 3.42% | 0.49% | 2.27% | 0.81% |

DAPI

| **LWD** | Avg copies/g DM | SD | SE |  | **SS** | Avg copies/g DM | SD | SE |
| --- | --- | --- | --- | --- | --- | --- | --- | --- |
| April | 4.18E+07 | 5.68E+06 | 2.54E+06 |  | April | 3.44E+07 | 4.67E+06 | 2.08E+06 |
| May | 4.56E+07 | 1.95E+06 | 8.69E+05 |  | May | 2.85E+07 | 2.77E+06 | 1.23E+06 |
| Sept | 4.10E+07 | 2.46E+06 | 1.10E+06 |  | Sept | 2.97E+07 | 4.99E+06 | 2.23E+06 |
| Nov | 4.43E+07 | 1.87E+06 | 8.34E+05 |  | Nov | 2.99E+07 | 1.28E+06 | 5.70E+05 |
| Dec | 4.80E+07 | 3.23E+06 | 1.44E+06 |  | Dec | 3.04E+07 | 1.62E+06 | 7.23E+05 |

**Flooding Experiment**

Physicochemical

|  |  |  |  |  |  |  |
| --- | --- | --- | --- | --- | --- | --- |
| **Site** | **Temp** (^o^C) | **pH** | **Turbidity** (NTU) | **DO** (mg/L) | **Specific Conductivity** (µS) | **Discharge** (L/s) |
|  |  |  |  |  |  |  |
| **Bench 1** | 27 | 6.69 | 11.8 | 2.38 | 652 | 3.4 |
| **Bench 2** | 26 | 7.09 | 12.1 | 1.8 | 662 | 3.5 |
| **Bench 3** | 26 | 7.16 | 11.5 | 2.82 | 659 | 3.7 |
| **Bench 4** | 26.5 | 7.1 | 10.9 | 1.98 | 671 | 3.1 |
| **Bench 5** | 26 | 7.34 | 12.4 | 2.42 | 669 | 3.4 |
|  |  |  |  |  |  |  |
|  |  |  |  |  |  |  |
| **Stream** | **% OM** | **Temp** (^o^C) | **pH** | **Turbidity** (NTU) | **DO** (mg/L) | **Specific Conductivity** (µS) |
|  |  |  |  |  |  |  |
| LWD | 7.04 | 26.3 | 7.08 | 11.74 | 2.28 | 662.6 |
|  | (± 0.37) | (± 0.20) | (± 0.11) | (± 0.26) | (± 0.18) | (± 3.44) |
|  |  |  |  |  |  |  |
|  |  |  |  |  |  |  |
|  |  |  |  |  |  |  |
| **Pipe** | **% OM** | **Temp** | **NO_3_^-^-N** (mg/L) |  |  |  |
| Flood | 6.27% | 25.9 | 2.41 |  |  |  |
|  | (± 0.10) | (± 0.10) | (± 0.08) |  |  |  |
| Dry | 15.34% | 26.3 | 2.36 |  |  |  |
|  | (± 0.23) | (± 0.14) | (± 0.06) |  |  |  |
|  |  |  |  |  |  |  |
|  |  |  |  |  |  |  |
| **Site** | **Pipe** | **Time** | **NO_3_^-^-N** (mg/L) | **% OM** |  |  |
| Bench 1 | Flood | Pre | 2.36±0.43 | 7.68±0.90 |  |  |
|  |  | 0 | 2.27±0.28 | 3.36±0.15 |  |  |
|  |  | 24 | 2.16±0.22 | 5.22±0.37 |  |  |
|  |  | 72 | 2.17±0.41 | 5.17±0.33 |  |  |
|  | Dry | Pre | 2.47±0.35 | 12.06±3.63 |  |  |
|  |  | 0 | 2.37±0.44 | 6.21±0.18 |  |  |
|  |  | 24 | 2.62±0.21 | 7.94±0.43 |  |  |
|  |  | 72 | 2.78±0.44 | 5.05±0.41 |  |  |
| Bench 2 | Flood | Pre | 2.28±0.11 | 4.86±1.56 |  |  |
|  |  | 0 | 2.36±0.09 | 3.51±0.29 |  |  |
|  |  | 24 | 2.41±0.23 | 6.61±0.23 |  |  |
|  |  | 72 | 2.42±0.18 | 6.18±0.36 |  |  |
|  | Dry | Pre | 2.53±0.15 | 18.29±1.52 |  |  |
|  |  | 0 | 2.24±0.10 | 5.93±0.14 |  |  |
|  |  | 24 | 2.45±0.12 | 5.55±0.99 |  |  |
|  |  | 72 | 2.62±0.32 | 6.39±0.31 |  |  |
| Bench 3 | Flood | Pre | 2.54±0.19 | 6.64±0.77 |  |  |
|  |  | 0 | 2.58±0.14 | 3.66±0.11 |  |  |
|  |  | 24 | 2.88±0.39 | 5.74±0.39 |  |  |
|  |  | 72 | 2.81±0.44 | 6.57±0.14 |  |  |
|  | Dry | Pre | 2.37±0.31 | 18.52±2.27 |  |  |
|  |  | 0 | 2.54±0.19 | 8.70±0.10 |  |  |
|  |  | 24 | 2.42±0.33 | 7.44±0.80 |  |  |
|  |  | 72 | 2.46±0.13 | 8.38±0.53 |  |  |
| Bench 4 | Flood | Pre | 2.22±0.08 | 4.78±0.26 |  |  |
|  |  | 0 | 2.61±0.37 | 3.06±0.40 |  |  |
|  |  | 24 | 2.49±0.15 | 5.59±0.21 |  |  |
|  |  | 72 | 2.44±0.18 | 5.66±0.38 |  |  |
|  | Dry | Pre | 2.35±0.34 | 12.40±0.78 |  |  |
|  |  | 0 | 2.06±0.13 | 6.09±0.13 |  |  |
|  |  | 24 | 2.53±0.14 | 5.03±0.76 |  |  |
|  |  | 72 | 2.67±0.20 | 4.91±0.57 |  |  |
| Bench 5 | Flood | Pre | 2.61±0.18 | 6.68±0.54 |  |  |
|  |  | 0 | 2.55±0.23 | 4.70±0.17 |  |  |
|  |  | 24 | 2.61±0.28 | 5.53±0.98 |  |  |
|  |  | 72 | 2.45±0.14 | 7.54±0.19 |  |  |
|  | Dry | Pre | 2.16±0.26 | 11.77±2.98 |  |  |
|  |  | 0 | 2.78±0.44 | 6.89±1.30 |  |  |
|  |  | 24 | 2.15±0.21 | 9.23±1.39 |  |  |
|  |  | 72 | 1.98±0.25 | 10.84±1.64 |  |  |
|  |  |  |  |  |  |  |

**Denitrification rate**

| MEANS | Flood | ± |  |  | Dry | ± |
| --- | --- | --- | --- | --- | --- | --- |
| Pre | 1.68E-02 | 8.30E-03 |  | Pre | 1.64E-02 | 9.30E-03 |
| 0 | 5.48E-02 | 9.50E-03 |  | 0 | 1.27E-02 | 4.81E-03 |
| 24 | 2.14E-02 | 7.41E-03 |  | 24 | 1.28E-02 | 4.13E-03 |
| 72 | 2.05E-02 | 7.68E-03 |  | 72 | 2.29E-02 | 5.69E-03 |

**Denitrifier gene abundance**

| Flood |  |  |  | Dry |  |
| --- | --- | --- | --- | --- | --- |
| Pre | 0.6% | 0.03% |  | 0.33% | 0.05% |
| 0 | 0.54% | 0.01% |  | 0.16% | 0.07% |
| 24 | 0.05% | 0.04% |  | 0.05% | 0.02% |
| 72 | 0.04% | 0.06% |  | 0.04% | 0.01% |
